# Preparation and electrochemical performance of VO_2_(A) hollow spheres as a cathode for aqueous zinc ion batteries

**DOI:** 10.1039/c9ra07340j

**Published:** 2019-10-30

**Authors:** Runxia Li, Xin Yu, Xiaofei Bian, Fang Hu

**Affiliations:** School of Materials Science and Engineering, Dongguan University of Technology Dongguan 523808 China 2018258@dgut.edu.cn; School of Materials Science and Engineering, Shenyang University of Technology Shenyang 110870 China hufang25@126.com

## Abstract

Rechargeable aqueous zinc ion batteries (ZIBs), owing to their low-cost zinc metal, high safety and nontoxic aqueous electrolyte, have the potential to accelerate the development of large-scale energy storage applications. However, the desired development is significantly restricted by cathode materials, which are hampered by the intense charge repulsion of bivalent Zn^2+^. Herein, the as-prepared VO_2_(A) hollow spheres *via* a feasible hydrothermal reaction exhibit superior zinc ion storage performance, large reversible capacity of 357 mA h g^−1^ at 0.1 A g^−1^, high rate capability of 165 mA h g^−1^ at 10 A g^−1^ and good cycling stability with a capacity retention of 76% over 500 cycles at 5 A g^−1^. Our study not only provides the possibility of the practical application of ZIBs, but also brings a new prospect of designing high-performance cathode materials.

## Abstract 2

VO_2_(A) hollow spheres exhibit superior zinc ion storage performance, large reversible capacity of 357 mA h g^−1^ at 0.1 A g^−1^, and good cycling stability with a capacity retention of 76% over 500 cycles at 5 A g^−1^

## Graphical abstract

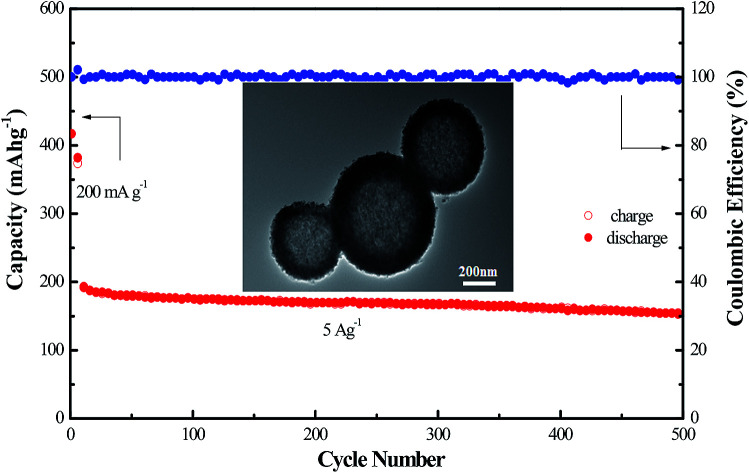

## 1. Introduction

Considering the increasingly serious energy shortage, climate change and other force majeure environmental factors, green renewable energy has become an active area of research. Secondary battery technologies afford a feasible way to store and convert the renewable energy sources; particularly, lithium-ion batteries account for half of the economic markets.^1–7^ However, the limited lithium reserves and insecurity make it tough for further scale-up. In the process of exploring alternative systems, rechargeable multivalent metal ion batteries, such as Al-ion battery, Ca-ion battery and Zn-ion battery, have shown potential application due to their abundant sources and environmental friendliness.^8–13^ In particular, rechargeable Zn-ion batteries will play a role in energy storage devices owing to the following advantages: (a) the high theoretical Zn metal anode capacity (820 mA h g^−1^), and the low oxidation–reduction potential of Zn^2+^/Zn (−0.76 V *vs.* standard 2H^+^/H_2_); (b) the low-cost (USD $2 kg^−1^) and abundant Zn sources, as well as the nontoxic and safe aqueous electrolyte; and (c) transfer of more charges during electrochemical reactions to provide more energy storage than the univalent batteries.^14–21^

The first aqueous ZIB was reported by Kang *et al.* using MnO_2_ as the Zn-storage cathode with zinc sulfate or nitrate aqueous electrolyte.^22–26^ Afterwards, open-framework materials were extensively investigated. To date, some other layered or tunnel-type vanadium-based compounds, such as V_2_O_5_·H_2_O,^27,28^ H_2_V_3_O_8_,^29^ Na_3_V_2_(PO_4_)_3_ (ref. 30) and VS_2_,^31^ have been studied, mainly due to their open-framework crystal structure and multivalent redox of vanadium ions. Alshareef *et al.* prepared Zn_3_V_2_O_7_(OH)_2_·2H_2_O nanowires, showing an initial discharge capacity of 148 mA h g^−1^ at the current density of 200 mA g^−1^ and the capacity retention is 68% after 300 cycles.^29^ Liang *et al.* reported Na_1.1_V_3_O_7.9_ nanoribbon/graphene, showing a capacity of 220 mA h g^−1^ at the current density of 300 mA g^−1^ with a capacity retention of 77% after 100 cycles.^32^

Herein, novel VO_2_(A) hollow spheres have been synthesized and tested as a cathode for rechargeable aqueous zinc ion battery. The as-prepared VO_2_(A) hollow spheres delivered superior electrochemical performance including large specific capacity (357 mA h g^−1^ at 0.1 A g^−1^), high rate capability (165 mA h g^−1^ at 10 A g^−1^) and good capacity retention (76% over 500 cycles at 5 A g^−1^).

## 2. Experimental

### 2.1 Synthesis

Initially, 2 mmol NH_4_VO_3_ was added to 45 mL distilled water and stirred at 80 °C for 10 min. After cooling to room temperature, 1 mmol HCl solution was added dropwise to the above solution and stirred slowly for 2 min. Then, 3 mL N_2_H_4_·H_2_O was added dropwise into the solution with continuous stirring until its color gradually changed from yellow to gray. The above suspension was poured in a 50 ml Teflon-lined autoclave and kept at 120 °C for 4 h. After the reaction, the samples were washed with distilled water and ethanol three times to remove any possible residues and dried in vacuum. Finally, the resultant products were heated under Ar gas in a tube furnace at the temperatures of 300 °C, 350 °C and 400 °C for 2 h with an increasing rate of 5 °C min^−1^, respectively.

### 2.2 Material characterizations

An X-ray diffractometer (XRD-7000, Shimadzu) with Cu Kα radiation (*λ* = 1.5406 Å) was used to investigate the structure of the materials. The morphology of the materials was studied by a Hitachi-4800 scanning electron microscope (SEM). High resolution transmission electron microscopy (HRTEM) was performed on a JEM-2100 PLUS high resolution transmission electron micro-scope. X-ray photoelectron spectroscopy (XPS) was conducted on an ESCALAB250 spectrometer using an Al Kα source. Total pore volumes of the materials were determined using the adsorbed volume at a relative pressure of 0.99. The multi-point Brunauer–Emmett–Teller (BET) surface area was estimated in the relative pressure range from 0.05 to 0.2.

### 2.3 Electrochemical experiments

Electrochemical tests were performed by using 2032-type coin cells, where a metallic zinc foil was used as the anode electrode. The working electrode was prepared as follows. First, 70 wt% active material and 20 wt% Super P conductive additive were mixed with alcohol for 1 h. Then, 10 wt% polytetrafluoroethylene (PTFE) was added into the above mixture and rolled to a film on a carbon paper. Lastly, the working electrode was dried overnight under air at 60 °C. The electrode mass loading on the carbon paper was about 1 mg. The electrolyte was 3 mol L^−1^ zinc trifluoromethanesulfonate (Zn(CF_3_SO_3_)_2_). Galvanostatic charge–discharge experiments were performed on a Land-2100 automatic battery tester. Cyclic voltammetry (CV) and galvanostatic intermittent titration technique (GITT) measurements were studied on a Bio-Logic VSP-300 multichannel.

## 3. Results and discussion

**Fig. 1:**
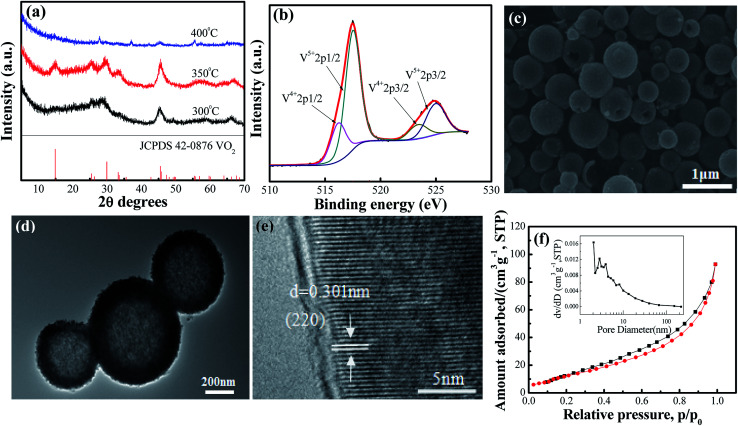
(a) XRD pattern of the as-prepared VO_2_(A) hollow spheres at 300 °C, 350 °C and 400 °C, respectively; (b) XPS survey spectrum of VO_2_(A) annealed at 350 °C samples; (c) SEM, (d) TEM and (e) HRTEM images of VO_2_(A) annealed at 350 °C; (f) Nitrogen adsorption–desorption isotherms and pore size distribution curves (inset) of VO_2_(A) annealed at 350 °C.

The crystal structures of VO_2_(A) hollow spheres were characterized by XRD, as shown in Fig. 1a. In the XRD pattern of VO_2_(A) annealed at 350 °C, five broad major reflections are located at 2*θ* = 14.9°, 25.5°, 29.9°, 33.3°, and 45.5°, which correspond to the (110), (102), (220), (212) and (330) crystal planes (JCPDS #42-0876, space group: P4_2_/*nmc*). No diffraction peaks of other V–O impurities were detected, indicating the successful preparation of VO_2_(A) with purity. In addition, we prepared vanadium dioxide samples at the temperatures of 300 °C and 400 °C, as shown in Fig. 1a, respectively. The sample annealed at 300 °C exhibited a slightly reduced crystallinity compared with the sample annealed at 350 °C. When the temperature was increased to 400 °C, the phase changed to VO_2_(M) (JCPDS #44-0252). Fig. 1b illustrates the high-resolution XPS spectra of the V element of the sample annealed at 350 °C. The V 2p_3/2_ in the VO_2_(A) could be resolved into two peaks at 516.2 and 517.5 eV, corresponding to V^4+^ and V^5+^.^33,34^ The intermediate oxidation peak between V^5+^ and V^4+^ is assigned to partial interaction with atmospheric oxygen because of the high surface area of the nanomaterials.^35,36^

In our experiment, the multi-step synthesis of VO_2_(A) hollow spheres can be described by the following reaction:

1 2VO_3_^−^ + N_2_H_4_·H_2_O + H_2_O → 2V(OH)_2_NH_2_↓ + 2O_2_↑

2
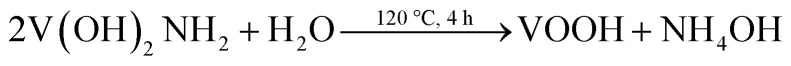
3
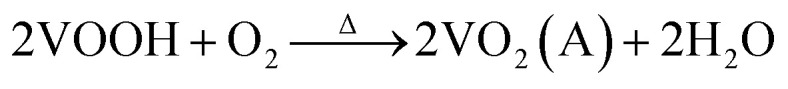


The reaction (1) shows that the V(OH)_2_NH_2_ suspension can be formed by adding N_2_H_4_·H_2_O with the solution color changing from yellow to gray. After being sealed in the autoclave at 120 °C for 4 h, the VOOH hollow spheres were obtained.^37,38^ For the third step, VOOH was oxidized to VO_2_(A) hollow spheres, which could be due to the small amount of oxygen gas existing in the Ar gas.

**Fig. 2:**
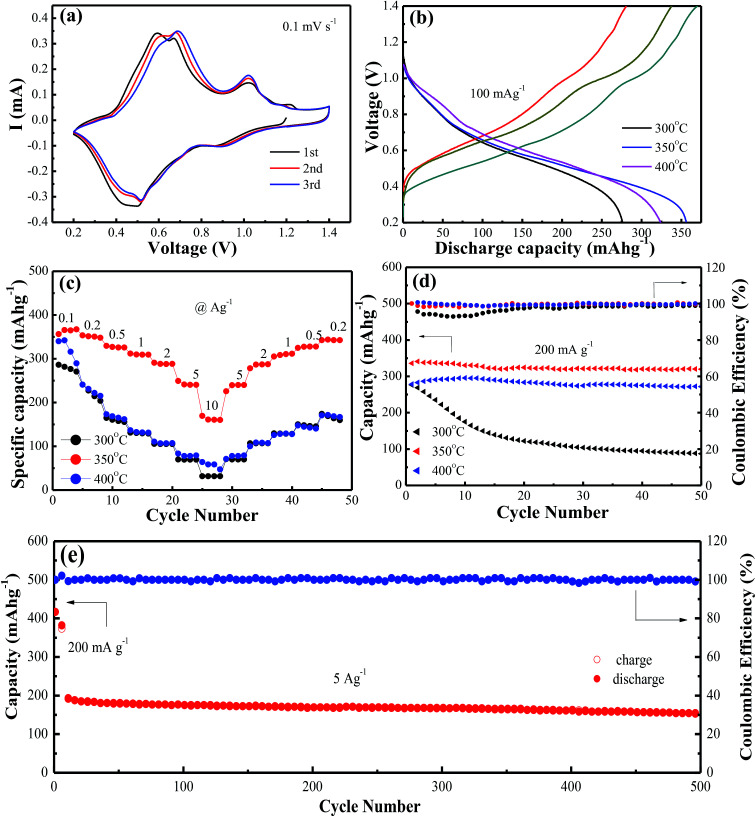
(a) CV profiles of VO_2_(A) annealed at 350 °C at the scan rate of 0.1 mV s^−1^; (b) The first discharge/charge profiles at the current density of 100 mA g^−1^; (c) rate performance; (d) cycling performance and coulombic efficiency at the current of 200 mA g^−1^ of VO_2_(A) hollow spheres at 300 °C, 350 °C and 400 °C, respectively; (e) cycling performance and coulombic efficiency of VO_2_(A) hollow spheres at the current density of 5 A g^−1^.

In order to observe the microscopic morphology of the VO_2_(A) sample, FESEM tests were performed, as shown in Fig. 1c. It could be seen that the size of the well-dispersed VO_2_(A) submicro-spheres is between 300–500 nm. From some broken spheres, VO_2_(A) spheres can be clearly observed with the hollow interior, which was further confirmed by the TEM image, as displayed in Fig. 1d. A strong contact between the dark outer shell and light inner could be readily observed for all the submicro-spheres, which is indicative of a uniform hollow framework. This structural peculiarity was vital for electrolyte flow into the internal space of the hollow spheres to improve the electrochemical properties. Fig. 1e shows the HRTEM images of VO_2_(A) hollow spheres. The lattice fringe with an interplanar distance of 0.301 nm corresponds to the (220) plane of the VO_2_(A) hollow sphere. Furthermore, the specific surface area and porosity of the as-prepared VO_2_(A) hollow spheres were measured by nitrogen gas adsorption–desorption isotherm, as shown in Fig. 2f. From the observed loop curve, it could be identified as a type IV isotherm with H3 hysteresis loop in the relative pressure range of 0.6–1.0 *P*/*P*_0_, indicating the existence of mesoporous and a high BET surface area up to 55.5 m^2^ g^−1^, with the pore size of 7.5 nm and pore volume of 0.16 cm^3^ g^−1^. The high surface area of VO_2_(A) hollow spheres was favorable for fast ion transport during the de/intercalation process, further facilitating the electrolyte uptake.

Fig. 2a shows cyclic voltammetry (CV) measurements at a scan rate of 0.1 mV s^−1^ to investigate the redox reaction of VO_2_(A) hollow spheres annealed at 350 °C in the range of 0.2–1.4 V. The initial three cycles of the CV profiles were almost overlapping, indicating the high reversibility of VO_2_(A) hollow spheres. The three pairs of easily identifiable reduction/oxidation peaks were positioned at 0.90/1.02 V, 0.52/0.68 V, and 0.44/0.58 V, respectively, attributing to a three step reaction associated with Zn^2+^ ion intercalation and extraction of the VO_2_ lattice.^39^Fig. 2b shows the initial charge/discharge curves of VO_2_(A) hollow spheres at 300 °C, 350 °C and 400 °C at the current density of 100 mA g^−1^, respectively. It was worth noting that the large initial charge/discharge capacities of 370/357 mA h g^−1^ at 350 °C had been received at this low current density, finally achieving a high coulombic efficiency of 104%. The VO_2_(A) products synthesized at 300 °C and 400 °C delivered the initial charge/discharge capacities of 338/324 and 280/276 mA h g^−1^, respectively, which were inferior to those of the VO_2_ sample synthesized at 350 °C. The rate capabilities of the above three VO_2_(A) hollow spheres had been investigated in Fig. 2c. The VO_2_(A) hollow spheres synthesized at 350 °C exhibited average discharge capacities of 357, 350, 325, 306, 285, 246, and 165 mA h g^−1^ at current densities of 0.1, 0.2, 0.5, 1.0, 2.0, 5.0, and 10.0 A g^−1^, respectively. In addition, when the current density returns to 0.2 A g^−1^, the reversible capacities could generally recover to the corresponding original values under various current densities. It was also noted that when the current densities increased to 5 and 10 A g^−1^, VO_2_ materials at 300 and 400 °C had almost no available capacity at these high current rates, which were much worse than those of VO_2_(A) materials at 350 °C. This could be due to the low crystallinity of the sample at 300 °C and small layer spacing (*d* = 3.20 Å) of VO_2_(M) at 400 °C compared to that of VO_2_(A) (*d* = 5.95 Å), both of which inhibit the fast Zn-ion insertion/extraction process. The cycling stability of VO_2_(A) material was tested at the current density of 0.2 A g^−1^, as shown in Fig. 2d. The specific capacities of the VO_2_(A) sample synthesized at 300 °C rapidly decreased after 50 cycles. In comparison, the VO_2_ sample synthesized at 350 °C and 400 °C was significantly improved with the retention of 94.1% and 92.5% of the maximum discharge capacity after 50 cycles. Lastly, the long-term cycle stability of the sample synthesized at 350 °C was investigated at the current density of 5 A g^−1^ with a pre-activation at 200 mA g^−1^ for the front five cycles, as shown in Fig. 2e. The initial discharge capacity at 5 A g^−1^ is about 202 mA h g^−1^. After 500 cycles, VO_2_(A) hollow spheres demonstrated a capacity of 154 mA h g^−1^ with a good retention of 76%, corresponding to a capacity decay rate of 0.096% per cycle.

In order to assess the capacitive and diffusion contributions to the total capacity of the VO_2_(A) electrode synthesized at 350 °C, CVs at the various scan rates from 0.1 to 1.0 mV s^−1^ were tested. Based on these scan data, the current (*i*) of CVs could comply with a power-law formula with the scan rate (*ν*) in the light of eqn (4):^40,41^

**Fig. 3:**
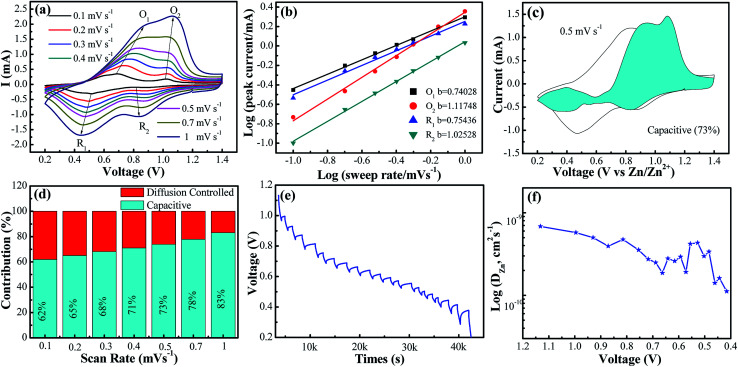
(a) CV curves at different scan rates from 0.1 to 1.0 mV s^−1^, respectively; (b) log(*i*)–log(*ν*) plots for specific peak currents at different scan rates to analysis the diffusion/capacity behaviors; (c) capacity separation analysis at 0.5 mV s^−1^. The blue-shaded data show the contribution to capacitive charge storage as a function of potential; (d) the contribution ratio of the capacitive capacities and diffusion-limited capacities for VO_2_(A) annealed at 350 °C at different voltage scan rates; (e) GITT date of VO_2_(A) annealed at 350 °C and (f) calculated Zn^2+^ diffusion coefficient.

4 *i* = *aν*^*b*^

where *i* is defined as the current density, *ν* is defined as the scan rate, and *a* and *b* are the adjustable parameters. When the *b*-value is 0.5, it suggests that the process is a diffusion-controlled process. When *b*-value is 1, it presents a capacitive-controlled process.^42^ The *b* values of the four redox peaks were calculated to be 0.74, 1.12, 0.75, and 1.03, respectively, as shown in Fig. 3b, suggesting that the charge storage process is mainly controlled by the capacitive behaviors. This led to a fast Zn^2+^ diffusion kinetics and high-rate performance. Furthermore, the capacity can be separated into capacitive-controlling (*k*_1_*ν*) and diffusion-controlling (*k*_2_*ν*^1/2^) at a specific scan rate according to the following eqn (5):^43^

5 *i* = *k*_1_*ν* + *k*_2_*ν*^1/2^

As depicted in Fig. 3c and d, with the incremental scan rate, the total charge came from the capacitive contribution gradually increasing from 62% to 83%, demonstrating that the capacitive-controlled contribution occupied an overwhelming proportion.^44^

Additionally, to further understand the details of the electrochemical behaviors of VO_2_ hollow spheres, the Zn^2+^ diffusion coefficients (*D*_Zn^2+^_) of the VO_2_(A) cathode were further determined by GITT. The GITT data were collected at a current density of 100 mA g^−1^ for 10 min and a rest interval of 30 min in Fig. 3e. The ionic diffusion coefficient in VO_2_(A) hollow sphere electrode can be calculated as follows:^45^6
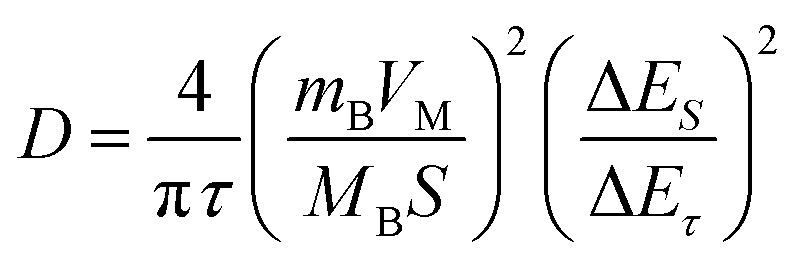
where *τ* is the constant current pulse time, *V*_m_, *m*_B_ and *M*_B_ are the molar volume, the mass loading and the molar mass of the inserted electrode material, respectively, *S* is the area interface between electrode material and electrolyte and Δ*E*_*τ*_ and Δ*E*_*S*_ represent the steady-state voltage change by the current pulse and voltage change during the constant current pulse, respectively. Fig. 3f illustrates that the Zn^2+^ diffusion coefficients of VO_2_(A) are in the range from 6.8 × 10^−10^ to 1.1 × 10^−10^ cm^2^ s^−1^, which is similar to that of VO_2_(D) hollow spheres.^46^ The high diffusion coefficient indicates the rapid Zn^2+^ movement in the VO_2_(A) hollow spheres.

**Fig. 4:**
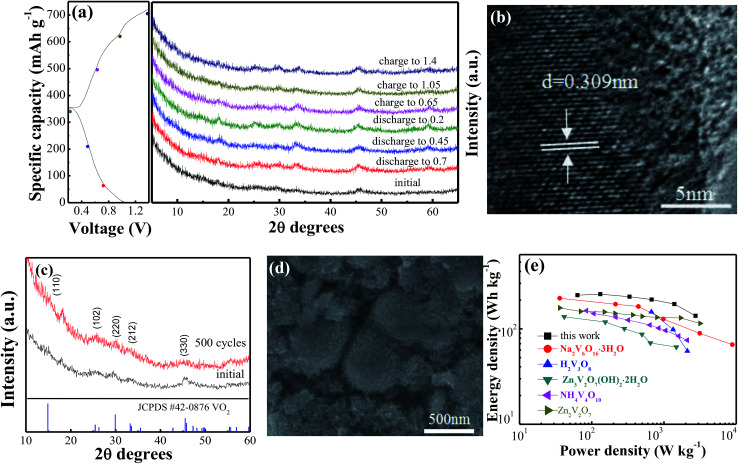
(a) *Ex situ* XRD arrays of VO_2_(A) annealed at 350 °C during the first discharge/charge process at the current density of 100 mA g^−1^; (b) HRTEM images of VO_2_(A) hollow spheres after discharged to 0.2 V; XRD (c) and SEM (d) of VO_2_(A) hollow spheres after 500 cycles at the current density of 5 A g^−1^; (e) Ragone plots of VO_2_(A) annealed at 350 °C and other electrodes for ZIBs.

*Ex situ* XRD was performed to investigate the evolution of the crystal structure during the discharge and charge process in the VO_2_(A) hollow spheres. Fig. 4a shows the *ex situ* XRD patterns of the VO_2_(A) electrode at different charge and discharge states in the first cycle. No significant shift of any diffraction peaks could be observed, indicating the stability of the structure of the VO_2_(A) electrode during the discharge–charge process. This can be attributed to the high capacitive behavior of the VO_2_(A) electrode in the ZIBs. Moreover, HRTEM analysis was carried out after discharging at 0.2 V, as shown in Fig. 4b. The interplanar distance (220) of the VO_2_(A) lattice expanded from 0.301 to 0.309 nm, corresponding to an increase in the c-parameter relative to the pristine VO_2_(A). The slight expansion of the lattice plane can be attributed to the large amounts of Zn^2+^ intercalation into the tunnels of VO_2_(A).

To provide further insight into the structural change of the VO_2_(A) cathode after the long-term cycling, XRD and SEM after 500 cycles at the current density of 5 A g^−1^ were performed, as displayed in Fig. 4c and d. The main diffraction peaks of VO_2_(A) at 2*θ* = 15.4°, 25.5°, 30.2°, 33.2° and 45.4° can still be observed, indicating a stable structure of the sample in the zinc ion intercalation/de-intercalation process. Moreover, the morphology of the VO_2_(A) hollow spheres is well maintained after cycling, confirming the structure stability of the material during the long-term cycling.

Fig. 4e displays the Ragone plots of the VO_2_(A) electrode along with reported cathode materials for ZIBs. Compared to other cathode materials such as Zn_3_V_2_O_7_(OH)_2_·2H_2_O,^47^ Zn_2_V_2_O_7_,^48^ H_2_V_3_O_8_,^35^ Na_2_V_6_O_16_·3H_2_O^49^ and NH_4_V_4_O_10_,^50^ VO_2_(A) exhibited a competitive energy density and power density (225 W h kg^−1^ at 63 W kg^−1^, respectively). When the power density was as high as 2736 W kg^−1^, the energy density remained at a high value of 137 W h kg^−1^. This demonstrates that VO_2_(A) hollow spheres are a promising cathode material for ZIBs.

## 4. Conclusions

In conclusion, VO_2_(A) hollow spheres with a high rate capability and large capacity have been investigated in this study. Satisfactorily, the VO_2_(A) hollow spheres delivered large specific capacity of 357 mA h g^−1^ at the current density of 0.1 A g^−1^, high rate performance with high rate capability of 165 mA h g^−1^ at 10 A g^−1^ and good cycling stability with a capacity retention of 76% over 500 cycles at 5 A g^−1^. Furthermore, the zinc storage mechanism and electrochemical dynamic properties of the VO_2_(A) cathode have also been analyzed systematically through DSCV, GITT, *ex situ* XRD and HRTEM measurements. The highly reversible de/intercalation reaction of bivalent Zn^2+^ can be attributed to the stable structure of VO_2_(A) hollow spheres, high capacitive-controlled contribution and good diffusion coefficient.

## Conflicts of interest

There are no conflicts to declare.

## Supplementary Material
